# Serum canine pancreatic-specific lipase concentrations in dogs with naturally occurring *Babesia rossi* infection

**DOI:** 10.4102/jsava.v86i1.1297

**Published:** 2015-08-13

**Authors:** Liza S. Köster, Jörg M. Steiner, Jan S. Suchodolski, Johan P. Schoeman

**Affiliations:** 1 Department of Companion Animal Clinical Studies, University of Pretoria, Onderstepoort, South Africa; 2 Department of Small Animal Clinical Sciences, Texas A&M University, United States

## Abstract

*Babesia rossi* is the cause of a highly virulent multisystemic disease with a variable outcome, which is a reliable model of systemic inflammatory response syndrome (SIRS). The objective of this study was to determine the concentration of canine pancreatic-specific lipase (cPL) in a population of dogs with naturally acquired *B. rossi* infection. In addition, the associations between serum cPL and death and SIRS status were examined. An observational study recruited 87 dogs diagnosed with *B. rossi* infection and serum cPL concentrations were measured daily until discharge or death. The median concentration of serum cPL was 124.0 µg/L (interquartile range: 51.0 µg/L – 475.5 µg/L) on admission (*n*= 87) and 145.5 µg/L (62.3 µg/L – 434.0 µg/L) on day two of hospitalisation (*n* = 40). Twenty-four dogs (28%) had a serum cPL concentration within the diagnostic range for pancreatitis (> 400 µg/L) at admission with 13 dogs (32.5%) presenting as such on the second day of hospitalisation. The median concentration of serum cPL in dogs with SIRS was 158 µg/L (interquartile range: 52.5 µg/L – 571.5 µg/L; *n* = 53), which was significantly higher than in those without SIRS (75 µg/L; 50.3 µg/L – 131.8 µg/L; *n* = 32) (*P* = 0.018). This study demonstrated that an unexpectedly high number of dogs diagnosed with naturally acquired canine babesiosis had a serum cPL concentration within the diagnostic range for acute pancreatitis and a significantly higher serum cPL concentration was found in dogs that were classified as having SIRS.

## Introduction

The prevalence of pancreatitis in a population of dogs with systemic inflammatory response syndrome (SIRS) is unknown. It has been suggested that, much like in humans, a large proportion of pancreatitis in dogs remains undiagnosed (Newman *et al.* 2004). Clinical signs associated with pancreatitis are not specific and thus the availability of a non-invasive commercial marker for use in a population of dogs with a variable prevalence of pancreatitis is clinically important.

Histopathology is considered definitive for the diagnosis of pancreatitis, but collecting a biopsy is quite invasive and the disease is often regionally localised (Newman *et al.* 2004). After the isolation of canine pancreatic-specific lipase (cPL), an enzyme-linked immunosorbent assay (ELISA) was developed and validated for diagnosing pancreatitis non-invasively in dogs (Steiner, Teague & Williams 2003; Steiner & Williams 2002). A commercially available assay for the measurement of cPL, namely Spec cPL^®^ (Idexx Laboratories, Westbrook) has been validated (Huth *et al.* 2010). This assay has demonstrated a moderate sensitivity (71% – 72%) with a cut-off of 200 µg/L, and an excellent specificity (80% – 100%) at a cut-off value of 400 µg/L (McCord *et al.* 2012; Neilson-Carley *et al.* 2011; Trivedi *et al.* 2011; Xenoulis & Steiner 2012). A positive cPL concentration as measured with Spec cPL has an excellent positive predictive value in a population likely to have acute pancreatitis and an excellent negative predictive value in a population with a low prevalence (McCord *et al.* 2012).

The virulent form of canine babesiosis, caused by *Babesia rossi* , is associated with various complications, including acute kidney injury, cerebral babesiosis, disseminated intravascular coagulation, hepatopathy, immune-mediated haemolytic anaemia, acute respiratory distress syndrome (ARDS), haemoconcentration, shock and pancreatitis (Defauw *et al.* 2012; Jacobson & Clark 1994; Lobetti & Jacobson 2001; Mohr, Lobetti & Van der Lugt 2000; Köster *et al.* 2009; Reyers *et al.* 1998; Schoeman 2009; Welzl *et al.* 2001).These complications are proposed to be the result of SIRS that is present in most cases of canine babesiosis, which could progress to multiple organ dysfunction syndrome (Welzl *et al.* 2001). The development of SIRS in dogs with a variety of diseases is associated with significantly higher mortality (18.8% versus 5.3%) (Okano *et al.* 2002).

The aims of this study were threefold. The first was to determine the serum cPL concentrations in a population of dogs with naturally acquired babesiosis due to *B. rossi* infection. Pancreatitis could potentially be the cause of some of the nonspecific gastrointestinal clinical signs in this disease. A concurrent diagnosis of acute pancreatitis would require additional therapeutic management considerations, including intravenous fluid therapy, anti-emetics and analgesics. The second aim was to compare serum cPL concentrations both in dogs that survived versus dogs that died, and in dogs with SIRS and those without based on the canine-specific criteria for SIRS (Okano *et al.* 2002). The final aim was to examine the association, if any, between serum cPL concentrations and clinical pathology and haematological parameters in dogs with babesiosis.

## Materials and methods

Serum samples and clinical data were collected for a prospective observational study conducted at the Onderstepoort Veterinary Academic Hospital (OVAH) to investigate the association of biomarkers and outcome in canine babesiosis in 2005 (protocol number V074/05). This study proposal was approved by the Animal Use and Care Committee and the Research Committee of the University of Pretoria. Owner consent was obtained upon enrolment of each dog.

This observational study reported on here was performed at OVAH, University of Pretoria. Patients were recruited from the Outpatient section and, if deemed critical enough to be hospitalised, were transferred to the intensive care facility and placed under the care of a clinician of the Small Animal Medicine Department. The haematology and clinical chemistry assays were performed at the Clinical Pathology Department, OVAH. Serum samples were shipped to the Gastrointestinal Laboratory, Texas A& M University, for Spec cPL analysis.

### Animals

A total of 98 dogs diagnosed with an acute *B. rossi* infection were recruited for this study. To be included in the study, dogs had to be diagnosed with canine babesiosis based on the morphological demonstration of intra-erythrocytic *Babesia* trophozoites on stained, thin, capillary blood films. Dogs that exhibited concurrent infections on clinical examination or those that had a history of being treated for an infection in the last 7 days were excluded from the study. Dogs infected with *Babesia vogeli* , or concurrent *Ehrlichia canis* or *Theileria* infection, and dogs euthanised for reasons other than poor prognosis were also excluded. A poor prognosis was based on previously established negative prognostic indicators, which included cerebral babesiosis, ARDS, hyperlactataemia and hypoglycaemia (Böhm *et al.* 2006; Goddard *et al.* 2013; Jacobson & Clark 1994; Keller *et al.* 2004; Köster *et al.* 2009; Nel *et al.* 2004; Schoeman, Rees & Herrtage 2007). In addition, dogs that did not have a complete medical record, including baseline haematology results, clinical data to classify the SIRS status, or insufficient stored serum to measure a serum cPL concentration with the Spec cPL assay were also excluded. All dogs received the standard treatment and supportive care for canine babesiosis as set out by the OVAH and determined by the primary clinician. The medical records were examined by one of the authors (L.S.K.) for clinical signs that could be consistent with, but not necessarily specific for, pancreatitis, namely vomiting, diarrhoea or abdominal pain.

### Polymerase chain reaction

Confirmation of infection with *B. rossi* was determined by a polymerase chain reaction reverse line blot (PCR RLB) hybridisation assay in batches, as described previously (Köster *et al.* 2009). Briefly, DNA was extracted from 200 µL of a whole-blood specimen. The QIAmp blood and tissue extraction kit (Qiagen, Hilden, Germany) was used for DNA extraction according to the manufacturer’s protocol. The *Babesia/Theileria/Hepatozoon* PCR was performed with primers RLB-F2 (5′-GAC ACA GGG AGG TAG TGA CAA G-3′) and RLB-R2 (biotin-5′-CTA AGA ATT TCA CCT CTG ACA GT-3′),which amplify a fragment of 460 bp – 540 bp from the 18S rRNA gene spanning the V4 region. The *Ehrlichia/Anaplasma* PCR was performed with the forward primer Ehr-F (5′-GGA ATT CAG AGT TGG ATC MTG GYT CAG-3′) and Ehr-R (biotin-5′- CGG GAT CCC GAG TTT GCC GGG ACT TYT TCT-3′), which amplify a fragment of 460 bp – 520 bp from the V1 hypervariable region of the 16S SSU rRNA gene. The conditions for the PCR included an initial step of 3 min at 42 °C, 10 min at 94 °C, then 10 cycles alternating through 94 °C (20 s), 67 °C (30 s) and 72 °C (30 s). The temperature for the annealing step was lowered by 2 °C after every second cycle (touchdown PCR). The reaction was then followed by 40 cycles of denaturation at 94 °C for 30 s, annealing at 57 °C for 30 s and extension at 72 °C for 30 s. RLB hybridisation was subsequently conducted on amplified products (*Babesia* ,* Theileria* ,* Hepatozoon* ,* Anaplasma* and *Ehrlichia* spp.) (also see Köster *et al.* 2009).

### Haematology and serum chemistry profile

Upon admission, a 2.5-mL blood sample was collected from the jugular vein of each dog, using a 22-gauge needle and a 3-mL syringe (Terumo, Somerset, United States). Blood samples were collected prior to any treatment. A portion of the sample was transferred into an EDTA tube and the remainder into a serum tube. The EDTA–whole-blood sample was submitted to the clinical pathology laboratory at OVAH for a complete blood count (Lasersyte, IDEXX, Westbrook). Serum samples were left to clot at room temperature and then centrifuged at 2100 g for 10 min. The serum was stored at -20 °C until later analysis. Stored serum was transported on ice to the Gastrointestinal Laboratory at Texas A& M University for Spec cPL measurement. The reference interval was set at < 200 µg/L and concentrations > 400 µg/L were considered consistent with pancreatitis; 200 µg/L – 400 µg/L was considered as the range for questionable diagnosis.

### Criteria for systemic inflammatory response syndrome

Dogs were classified as meeting the criteria for SIRS if two or more of the following parameters had been met, as described by Okano *et al.* (2002):

- a white cell count exceeding 12 000/µL or less than 4000/µL, or presence of ≥ 10% immature or band cells
- a rectal temperature < 37.8 °C or > 39.7 °C
- a heart rate of at least 160 beats per minute
- a respiratory rate of ≥ 40 breaths per minute.

### Statistical analysis

Data were analysed using a commercial statistical software package (SPSS Statistics 21, IBM, New York). Data were analysed for normality using the one-sample Kolmogorov–Smirnov test. Serum cPL concentrations were compared between dogs with SIRS and those without SIRS, as well as between survivors and non-survivors, using the Mann–Whitney *U* -test. A Spearman’s rank order correlation (*r*_s_) was used to examine the associations between:

- serum cPL and clinical parameters, including rectal temperature, heart rate and respiratory rate
- serum cPL and haematological parameters, including immature neutrophil count and percentage of band cells
- serum cPL and clinical pathology parameters, including amylase, lipase, alanine aminotransferase and creatinine
- serum cPL and endocrine parameters, thyroid stimulating hormone, thyroxin (T_4_), free thyroxin (fT_4_), endogenous adrenocorticotropic hormone (ACTH) and cortisol.

A *P* -value of < 0.05 was considered statistically significant.

## Results

Of the 98 dogs recruited for this study, 11 were excluded for the following reasons: incomplete medical records (*n* = 1), insufficient clinical or laboratory data (*n* = 4), insufficient stored serum (*n* = 3) or spillage of the stored serum during transportation to the laboratory in Texas (*n* = 3). Therefore, 87 dogs were included in the final analysis, of which 54 were male and 33 were female. The median body mass was 16.4 kg (range: 1.9 kg – 54.3 kg) and the median age was 14 months (range: 2–156 months). A total of six dogs (6.9%) died as a result of the infection or were euthanised based on a poor prognosis. When the historical and clinical signs recorded in the medical records were examined, 17 dogs experienced vomiting, 5 dogs had diarrhoea, and 4 dogs exhibited abdominal pain. Two dogs presented with both vomiting and diarrhoea, three with vomiting and abdominal pain, and one dog exhibited all three clinical signs. Therefore, either vomiting or diarrhoea was seen as the only gastrointestinal sign in 12 and 3 dogs, respectively. This analysis shows that 20 of the 87 dogs (22.9%) had clinical signs that could be consistent with pancreatitis, with five of this subgroup presenting with a serum cPL concentration > 400 µg/L.

The median and interquartile range concentration of serum cPL was 124.0 µg/L (51.0 µg/L – 475.5 µg/L) on admission (*n* = 87), and 145.5 µg/L (62.3 µg/L – 434.0 µg/L) on day two of hospitalisation (*n* = 40). Of the dogs with acute babesiosis, 24 (28%) were classified as positive for pancreatitis based on the recommended cut-off value (> 400 µg/L) when tested at admission. On the second day of hospitalisation, an abnormal serum cPL concentration was measured in 13 dogs (32.5%).

**FIGURE 1:**
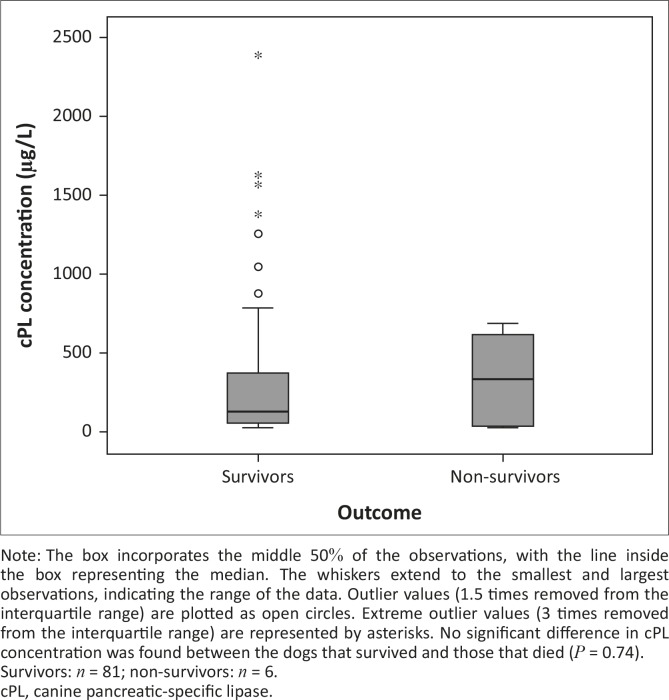
A boxplot depicting the canine pancreatic-specific lipase concentration at admission (day 1) in dogs presenting with naturally acquired canine babesiosis and categorised according to outcome.

**FIGURE 2:**
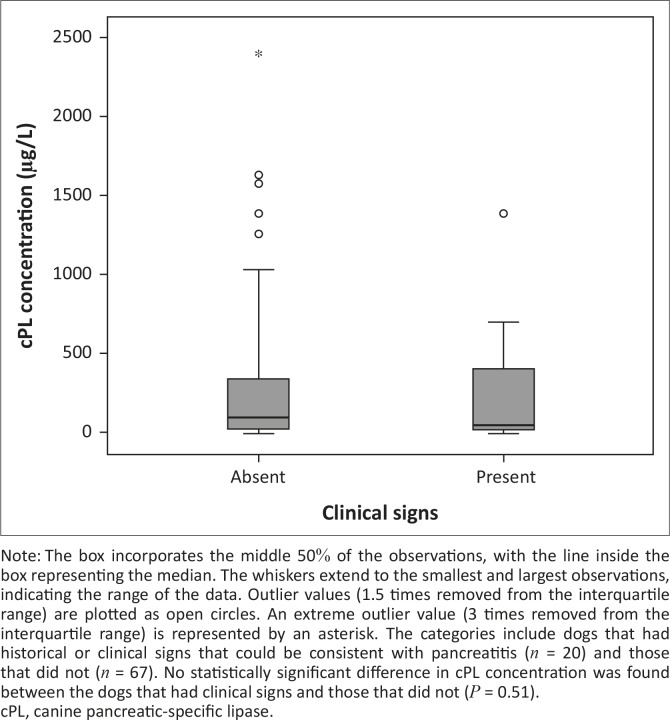
A boxplot depicting the canine pancreatic-specific lipase concentration at admission (day 1) in dogs presenting with naturally acquired canine babesiosis, grouped according to presence of clinical signs.

**FIGURE 3:**
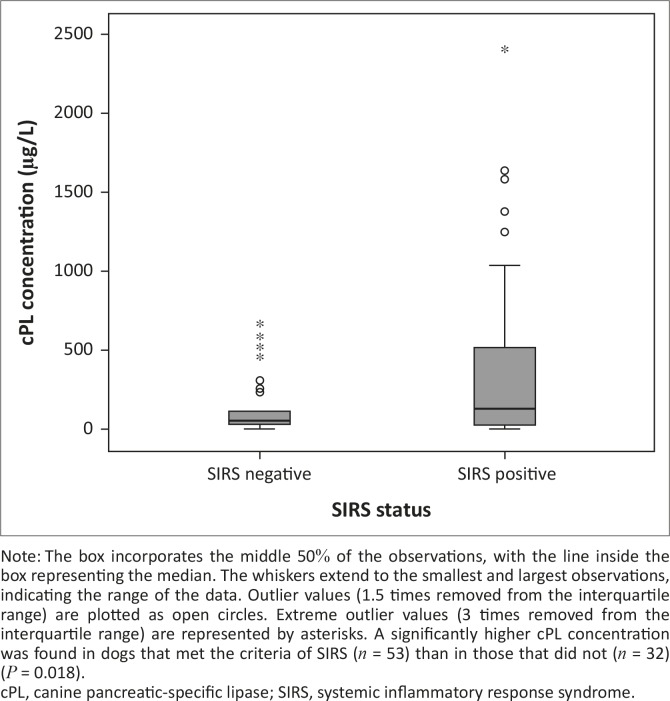
A boxplot depicting the canine pancreatic-specific lipase concentration at admission (day 1) in dogs presenting with naturally acquired canine babesiosis, grouped according to their status with regard to systemic inflammatory response syndrome.

Three of the six dogs that died had a serum cPL concentration > 400 µg/L. Serum cPL concentrations were not significantly different between non-survivors and survivors (*P* = 0.74; Figure 1), or between dogs with pancreatitis-related clinical signs and those without (*P* = 0.51; Figure 2). The median concentration of serum cPL in dogs with SIRS was 158 µg/L (interquartile range: 52.5 µg/L – 571.5 µg/L; *n* = 53), which was significantly higher than in dogs without SIRS (75 µg/L; interquartile range: 50.3 µg/L – 131.8 µg/L; *n* = 32) (*P* = 0.018; Figure 3).

Clinically, the concentration of serum cPL as measured with the Spec cPL assay was significantly positively correlated with the immature neutrophil count (*r*_s_ = 0.34; *P* < 0.01), the percentage of neutrophilic band cells (*r*_s_ = 0.32; *P* = 0.01), amylase activity (*r*_s_ = 0.40; *P* < 0.01) and lipase activity (*r*_s_ = 0.78; *P* < 0.01). Serum cPL concentration was significantly negatively correlated with rectal temperature (*r*_s_ = -0.22;* P* = 0.04) and fT_4_ concentration (*r*_s_ = -0.23, *P* = 0.04). Serum cPL concentration was not significantly correlated with mature neutrophils or any other measured clinical, biochemical or endocrinological parameters (Table 1).

**TABLE 1:** The results of the Spearman’s rank order correlation (*r*_s_) between canine pancreatic-specific lipase concentration and various clinical, haematological, biochemical and endocrine parameters in dogs with naturally acquired canine babesiosis caused by *Babesia rossi* infection (*P* ≤ 0.05).

Parameter	rs value	P-value
Rectal temperature	−0.22	0.04*
Heart rate	−0.02	0.98
Respiratory rate	0.02	0.85
Mature neutrophil count	−0.28	0.8
Immature neutrophil count	0.29	< 0.01*
Band cell percentage	0.28	0.01*
Amylase	0.40	< 0.01*
Lipase	0.74	< 0.01*
Creatinine	−0.03	0.82
ALT	0.16	0.15
ACTH	−0.07	0.55
Cortisol	0.2	0.06
TSH	−0.06	0.6
T4	−0.18	0.1
fT4	−0.23	0.04*

ACTH, adrenocorticotropin hormone; ALT, alanine transaminase; TSH, thyroid stimulating hormone; T_4_, total thyroxin; fT_4_, free thyroxin.

*, Indicate significant correlations.

## Discussion

To the authors’ knowledge, this study is the first to demonstrate an association between SIRS and abnormal pancreatic-specific lipase concentration. Notably, dogs with babesiosis that presented with SIRS had a significantly higher serum cPL concentration than dogs that did not meet the criteria for SIRS. As a result, a strong association was found between serum cPL and some of the parameters of SIRS, namely low rectal temperature, immature neutrophil count and the percentage of band cells. These parameters have previously been reported to be the most strongly weighted parameters of SIRS outcome (Okano *et al.* 2002). Another reason for this association could be the corollary that pancreatitis arising from acute babesiosis is a cause of SIRS. An increased serum cPL concentration in canine babesiosis could be related to a reduced glomerular filtration rate, which has been described previously in this infectious disease (Lobetti & Jacobson 2001). It should, however, be noted that in another study (Steiner, Finco & Williams 2010), no clinically significant increases in specific pancreatic lipase were found in dogs with experimentally induced renal failure, suggesting that glomerular filtration rate has no effect on serum cPL concentration. Similar to the study of Steiner *et al.* (2010), our study did not show any significant correlation between serum cPL and creatinine concentrations (*P* = 0.82) and thus glomerular filtration rate was thought to be an unlikely contributing factor. A previous study investigating the acute virulent form of canine babesiosis found that 87% of complicated cases met the requirements for SIRS (Welzl *et al.* 2001). In comparison, based on the criteria for canine SIRS outlined previously by Okano *et al.* (2002), our study found the prevalence of SIRS to be 61%. The reason for the lower prevalence is that all forms of babesiosis (i.e. both uncomplicated and complicated forms) were included in the study.

The current study detected a serum cPL concentration within the diagnostic range associated with acute pancreatitis in a number of dogs diagnosed with the peracute virulent form of canine babesiosis, caused by *B. rossi* , during the first 2 days of hospitalisation. A study examining serum cPL concentration in dogs with ehrlichiosis found 20% of dogs with natural infection had results consistent with the diagnosis of acute pancreatitis; however, none of the dogs displayed specific gastrointestinal signs, only nonspecific clinical signs, including anorexia and depression (Mylonakis *et al.* 2014). The virulent form of canine babesiosis shares many similarities with human malaria caused by *Plasmodium falciparum* and therefore the naturally acquired infection in dogs has been proposed as an animal model for this disease (Reyers *et al.* 1998). A number of case reports describe acute pancreatitis as a complication of this type of malaria in humans (Johnson, DeFord & Carlton 1977; Kumar, Jain & Vikas 2010; Mandal *et al.* 2011; Mohapatra & Gupta 2011; Sarma & Kumar 1998; Seshadri *et al.* 2008; Thapa, Mallick & Biswas 2010; Trowers *et al.* 1992).

Moreover, acute pancreatitis has previously been identified as a potential complication of canine babesiosis (Mohr *et al*. 2000); however, very few of the dogs had abdominal ultrasound performed. Postmortem and histological examination of the pancreas were performed on the dogs that died. Of those, 18 (24%) met the criteria of having serum amylase activity of > 3600 U/L or lipase activity of > 650 U/L; histopathological changes confirming acute pancreatitis were found in four dogs (Mohr *et al.* 2000). Ostensibly, the prevalence of pancreatitis in this study (28%), as determined by Spec cPL, was similar to this retrospective study (Mohr *et al.* 2000) using amylase and lipase activities (24%) as diagnostic criteria. However, closer inspection revealed that Mohr *et al.* (2000) tested a highly selected population, namely cases with complicated babesiosis admitted to intensive care and sampled for varying periods after admission. When the date of admission is considered as a criterion in that study, the prevalence drops to 2.6%.

The measurement of serum amylase and lipase activities as markers for acute pancreatitis has several limitations: (1) serum amylase and lipase activities increase with renal failure, (2) increased serum activities are not specific for pancreatitis and (3) low serum activity cannot rule out the disease (Mia, Koger & Tierney 1978; Strombeck, Farver & Kaneko 1981). Serum lipase activity has been reported to have a poor specificity for pancreatitis (42.9%), with sensitivity for severe pancreatitis as high as 71% (Strombeck *et al.* 1981; Trivedi *et al.* 2011). The pancreas was found not to be the exclusive organ of origin of amylase, lipase and iso-amylase activities, because these parameters failed to decline after experimental pancreatectomy in the dog (Simpson *et al.* 1991). Subsequently there have been a number of endeavours to isolate specific serological markers of pancreatic inflammation (Mansfield, Jones & Spillman 2003; Mansfield, Watson & Jones 2011; Ruaux & Atwell 1999). It was not until pancreatic lipase had been purified from canine pancreatic tissue that both a radioimmunoassay and later an ELISA were validated for the measurement of pancreatic lipase immunoreactivity (Steiner *et al.* 2003; Steiner & Williams 2002, 2003). cPL is considered to have acceptable test accuracy and can be used for diagnosing pancreatitis non-invasively both by the Spec cPL assay and a point-of-care test, SnapcPL (McCord *et al.* 2012; Steiner *et al.* 2008; Trivedi *et al.* 2011).

Diagnosis of pancreatitis in dogs with babesiosis is hindered by mild to moderate forms of pancreatitis not being associated with clinical signs or by clinical signs overlapping with those of babesiosis; yet even mild or moderate pancreatitis may contribute overall to morbidity and mortality. Carney *et al.* (2011) proposed that population-specific reference ranges need to be created to improve sensitivity of detecting pancreatitis in dogs with chronic stable disease versus dogs with clinical signs ascribable to severe acute pancreatitis. The specificity of serum cPL concentration, with a cut-off value > 400 µg/L, was 97.5% (Neilson-Carley *et al.* 2011; Newman *et al.* 2004). Thus, at a cut-off of 400 µg/L, few false-positive results were expected in this study.

The current study did not show an association between serum cPL and mortality. Thus, it can be assumed that the abnormally elevated serum cPL in dogs with babesiosis is not a risk factor for death. However, the lack of differences in serum cPL concentration between survivors and non-survivors is most likely due to the low mortality rate in this study and in babesiosis in general, when treated appropriately. A similar lack of significant difference was found when C-reactive protein concentrations were compared between survivors and non-survivors in dogs naturally infected with *B. rossi* , despite it being a significant prognosticator in a number of other canine diseases (Köster *et al.* 2009). However, several studies have reported on a number of indices as predictors of mortality in canine babesiosis, such as a higher capillary and venous parasitaemia and a collapsed state, hypoglycaemia upon admission, hyperlactataemia that fails to decline by more than 50% after 24 hours, coagulopathy and specific organ involvement (Böhm *et al.* 2006; Goddard *et al.* 2013; Jacobson & Clark 1994; Keller *et al.* 2004; Nel *et al.* 2004). In addition, in an earlier study, mortality was shown to be associated with endocrine parameters, with significantly higher cortisol and ACTH concentrations and lower T_4_ and fT_4_ concentrations occurring in the group of dogs that died compared with the survivors (Schoeman *et al.* 2007). Our study found a significant negative correlation between serum cPL and fT_4_ concentrations, which could be explained by the euthyroid sick syndrome.

The limitations of the current study included the lack of abdominal ultrasound imaging, which was not consistently performed. Also, as pathology records were not available for all non-survivors, pancreatitis could not be confirmed. It can be argued that the increased serum cPL concentration could have been a result of leakage due to babesiosis-related vasculitis rather than active pancreatitis. Ideally, a prospective study should be designed, using a homogeneous model of SIRS, to measure cPL concentration, with dogs undergoing a complete abdominal ultrasound and complete postmortem examinations of all non-survivors. Lastly, to consider the parasite role in pancreatic inflammation, cPL concentration should be compared between dogs with SIRS due to canine babesiosis and a non-infectious, inflammatory condition.

## Conclusion

A number of dogs with the peracute virulent form of canine babesiosis had a cPL concentration consistent with the diagnosis of acute pancreatitis (> 400 µg/L). A smaller percentage of these dogs displayed clinical signs that could be consistent with pancreatitis. A significantly higher serum cPL concentration was found in dogs that were classified as having SIRS. Immature neutrophils and band cells were positively correlated with serum cPL concentration and acute pancreatitis should be suspected when these parameters are elevated. cPL is strongly associated with SIRS as clinical outcome, which could be interpreted as a clinical prognostic factor. Consequently, a larger study population, consisting of a sufficient number of dogs that died and on which necropsy are performed, will be needed to elucidate the role of pancreatic inflammation in canine babesiosis and the specificity of this test in dogs with SIRS.
